# Additional radiotherapy to breast‐conserving surgery is an optional treatment for de novo stage IV breast cancer: A population‐based analysis

**DOI:** 10.1002/cam4.3751

**Published:** 2021-02-14

**Authors:** Jun Wang, Shi‐Ping Yang, Ping Zhou, Chen‐Lu Lian, Jian Lei, Li Hua, Zhen‐Yu He, San‐Gang Wu

**Affiliations:** ^1^ Department of Radiation Oncology The First Affiliated Hospital of Xiamen University Xiamen People’s Republic of China; ^2^ Department of Radiation Oncology Hainan General Hospital (Hainan Affiliated Hospital of Medical University) Haikou Hainan People’s Republic of China; ^3^ Department of Obstetrics and Gynecology The First Affiliated Hospital of Xiamen University Xiamen People’s Republic of China; ^4^ Department of Radiation Oncology Sun Yat‐sen University Cancer Center State Key Laboratory of Oncology in South China Collaborative Innovation Center of Cancer Medicine Guangzhou People’s Republic of China

**Keywords:** breast‐conserving surgery, mastectomy, metastatic breast cancer, radiotherapy, survival

## Abstract

**Background:**

We aim to assess the value of locoregional treatment (LRT) including breast‐conserving surgery (BCS), mastectomy (MAST), and radiotherapy (RT) in patients with de novo stage IV breast cancer.

**Methods:**

Patients with de novo stage IV breast cancer were retrospectively identified from the Surveillance, Epidemiology, and End Results database between 2004 and 2014. Kaplan‐Meier analysis, log‐rank tests, propensity score matching (PSM), and the multivariate Cox proportional model were used for statistical analysis.

**Results:**

A total of 5798 patients were identified including 849 (14.6%), 763 (13.2%), 2338 (40.3%), and 1848 (31.9%) who received BCS alone, BCS+RT, MAST alone, and MAST+RT, respectively. The proportions of receiving BCS decreased from 35.9% in 2004 to 26.2% in 2014 (*p* = 0.002), and the probability of patients receiving MAST increased from 64.1% in 2004 to 74.8% in 2014 (*p* = 0.002). Before PSM, there was a significant difference in breast cancer‐specific survival (BCSS) among the treatment arms. Patients who received RT had better BCSS, the 5‐year BCSS was 40.5%, 52.3%, 41.5%, and 47.7% in patients treated with BCS alone, BCS+RT, MAST alone, and MAST+RT, respectively (*p* < 0.001). In the PSM cohort, patients treated with BCS alone had lower 5‐year BCSS compared to those treated with BCS+RT (43.9% and 52.1%, *p* = 0.002). However, there were comparable 5‐year BCSS between BCS+RT and MAST alone groups (51.3% and 50.1%, *p* = 0.872), and BCS+RT and MAST+RT cohorts (51.5% and 55.7%, *p* = 0.333). Similar results were confirmed in multivariate analysis.

**Conclusions:**

Postoperative RT improves BCSS in patients with de novo stage IV breast cancer, and BCS+RT shows a non‐inferior outcome compared to MAST+RT. BCS+RT may be the optimal local management of de novo stage IV breast cancer.

## Graphical abstract

In this population‐based retrospective study, we aim to compare the differences on survival between breast‐conserving surgery (BCS) and mastectomy (MAST) in de novo stage IV breast cancer. In addition, we further analyzed the effect of additional radiotherapy (RT) on survival outcomes in this patient subset. Our results showed that postoperative RT improves BCSS in patients with de novo stage IV breast cancer, and BCS+RT shows non‐inferior outcome compared to MAST+RT. BCS+RT may be the optimal local management of de novo stage IV breast cancer.

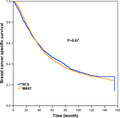

## 1 INTRODUCTION

Approximately 2 million women were newly diagnosed with breast cancer in 2018 worldwide, accounting for 11.6% of all cancer types, and breast cancer is the leading cause of cancer death in females.^1^ De novo stage IV breast cancer, which is defined by metastasis from the breast and axilla to distant sites, was approximately 3.6%–6% of all newly diagnosed breast cancer.^2^, ^3^ Bone (66.3%), lung (30.3%), liver (26.1%), and brain (7.3%) were the most common sites of distant metastasis.^4^ The clinical outcome in patients with de novo stage IV breast cancer was significantly poor with median survival ranging from 16 to 29 months.^5^, ^6^, ^7^ Systemic therapy was a mainstay of treatment in patients with de novo stage IV breast cancer.^5^, ^8^, ^9^, ^10^, ^11^ Locoregional treatments (LRTs), including surgery or radiotherapy (RT), are recommended just for relieving symptoms, and whether LRT should be administrated to primary tumor remains controversial. Two large prospective studies of local surgery have yielded conflicting results^7^, ^12^; however, most of the current retrospective studies showed a survival advantage with local surgery to the primary tumor.^13^, ^14^, ^15^, ^16^

RT is still deemed to be one of the significant ways of LRT, whereas related studies are rare and inconclusive compared with surgery. A retrospective study from France regarded the effect of RT in de novo stage IV breast cancer, and they found that RT alone provided comparable metastasis progression‐free survival and overall survival (OS) rates compared to the combination of RT and surgery.^17^ However, another population‐based study including 2207 patients with de novo stage IV breast cancer showed that post‐surgery RT increased breast cancer‐specific survival (BCSS) rates compared to surgery alone.^18^ Given the controversy, several prospective randomized trials are in progress to sort out this concept.^19^, ^20^, ^21^ However, these trials all focused on the value of surgery in de novo stage IV breast cancer. To the best of our knowledge, the survival benefit of RT has been rarely elucidated in the prospective study; therefore, it is worth exploring the value of postoperative RT in this disease. In addition, the optimal procedure of surgical approaches and whether additional RT after surgery has survival benefit remain unclear. In this population‐based retrospective study, we aim to compare the differences in survival between breast‐conserving surgery (BCS) and mastectomy (MAST) in de novo stage IV breast cancer. In addition, we further analyzed the effect of postoperative RT on survival outcomes in this patient subset.

## 2 METHODS AND MATERIALS

### 2.1 Database source and patient's collection

The Surveillance, Epidemiology, and End Results (SEER) database was used to identify patients. The SEER database is a population‐based, open‐access resource collecting information on cancer prevalence, management, and survival for approximately 28% of the United States population.^22^ We included patients who met the following criteria: (a) female patients with newly diagnosed metastatic breast cancer from 2004 to 2014; (b) receiving systemic chemotherapy and local therapy including BCS and MAST; and (c) availability of data on age, race/ethnicity, tumor (T) stage, nodal (N) stage, estrogen receptor (ER), progesterone receptor (PR), surgical approaches, and RT records. Patients without positive pathological diagnosis and receiving non‐beam irradiation were excluded. Non‐beam irradiation was defined as RT technologies including radioactive implants and radioisotopes. We obtained the access to the SEER database, and using the data was exempt from the approval of the Institutional Review Board for not involving any private information.

### 2.2 Variables

The following baseline variables were identified: age at diagnosis (<65 years, ≥65 years), race/ethnicity (non‐Hispanic White, non‐Hispanic Black, Hispanic, and Other), histology (invasive ductal carcinoma [IDC], invasive lobular carcinoma [ILC], and other), grade (well‐differentiated, moderately differentiated, and poorly differentiated/undifferentiated), T stage (T1, T2, T3, and T4), N stage (N0, N1, N2, and N3), ER status (ER positive and ER negative), PR status (PR positive and PR negative), treatment procedures (BCS alone, BCS+RT, MAST alone, and MAST+RT). The TNM stage was defined using the 7th edition of the American Joint Committee on Cancer staging system.^23^ The primary objective of this study was BCSS, defined as the time interval from diagnosis to death from breast cancer.

### 2.3 Statistical analysis

Chi‐square test was used to analyze the differences in demographic and clinicopathological characteristics among the treatment groups. Survival curves were delineated with the Kaplan‐Meier analysis and compared with the log‐rank test. Propensity score matching (PSM) was performed using 1:1 nearest neighbor matching to balance the distribution of most demographic and clinical characteristics including age at diagnosis, race/ethnicity, histology, grade, T stage, N stage, ER status, and PR status. Cox proportional hazards multivariable regression was used to calculate the independent risk factors of BCSS. All statistical results were conducted using SPSS statistical software (version 22.0, IBM Corporation, Armonk, NY, USA). A *p* < 0.05 (two‐tailed) was considered statistically significant.

## 3 RESULTS

### 3.1 Patient characteristics

In totality, 5798 patients were identified with a median age of 55 years (range, 19–94 years). The patient baseline characteristics are shown in Table 1. All of the patients received systematic chemotherapy. Majority of the patients were non‐Hispanic White (62.1%), IDC (77.2%) subtype, poor differentiation/undifferentiated (63.9%), and ER positive (62.3%) disease. In addition, 48.8% and 45.5% of the patients experienced T3‐4 and N2‐3 disease, respectively. With regarding to LRT, 14.6% (n = 849), 13.2% (n = 763), 40.3% (n = 2338), and 31.9% (n = 1848) of patients were treated with BCS alone, BCS+RT, MAST alone, and MAST+RT, respectively.

**TABLE 1:** Baseline characteristics of the patients with de novo stage IV breast cancer before PSM

Variables	N (%)	BCS	BCS+RT	MAST	MAST+RT	*p*
Age (years)						
<65	4415 (76.1)	613 (72.2)	617 (80.9)	1719 (73.5)	1466 (79.3)	<0.001
≥65	1383 (23.9)	236 (27.8)	146 (19.1)	619 (26.5)	382 (20.7)	
Race/ethnicity						
Non‐Hispanic White	3603 (62.1)	542 (63.8)	475 (62.3)	1438 (61.5)	1148 (62.1)	0.361
Non‐Hispanic Black	1011 (17.4)	148 (17.4)	117 (15.3)	433 (18.5)	313 (16.9)	
Hispanic	701 (12.2)	103 (12.1)	102 (13.4)	273 (11.7)	223 (12.1)	
Other	483 (8.3)	56 (6.6)	69 (9)	194 (8.3)	164 (8.9)	
Pathological subtype						
IDC	4474 (77.2)	681 (80.2)	638 (83.6)	1771 (75.7)	1384 (74.9)	<0.001
ILC	373 (6.4)	52 (6.1)	34 (4.5)	158 (6.8)	129 (7)	
Other	951 (16.4)	116 (13.7)	91 (11.9)	409 (17.5)	335 (18.1)	
Grade						
Well differentiated	284 (4.9)	58 (6.8)	47 (6.2)	104 (4.4)	75 (4.1)	0.001
Moderately differentiated	1807 (31.2)	245 (28.9)	263 (34.5)	701 (30)	598 (32.4)	
Poorly differentiated/ undifferentiated	3707 (63.9)	546 (64.3)	453 (59.3)	1533 (65.6)	1175 (63.5)	
Tumor stage						
T1	812 (14)	218 (25.7)	208 (27.3)	247 (10.6)	139 (7.5)	<0.001
T2	2155 (37.2)	404 (47.5)	381 (49.9)	827 (35.4)	543 (29.4)	
T3	1049 (18.1)	89 (10.5)	76 (10)	485 (20.7)	339 (21.6)	
T4	1782 (30.7)	138 (16.3)	98 (12.8)	779 (33.3)	767 (41.5)	
Nodal stage						
N0	979 (16.9)	241 (28.4)	202 (26.5)	382 (16.3)	154 (8.3)	<0.001
N1	2182 (37.6)	327 (38.5)	297 (38.9)	865 (37)	693 (35.7)	
N2	1188 (20.5)	135 (15.9)	121 (15.9)	490 (21)	442 (23.8)	
N3	1449 (25)	146 (17.2)	143 (18.7)	601 (25.7)	559 (30.2)	
ER status						
Negative	2183 (37.7)	347 (40.9)	256 (33.6)	945 (40.4)	635 (34.4)	<0.001
Positive	3615 (62.3)	502 (59.1)	507 (66.4)	1393 (59.6)	1213 (65.6)	
PR status						
Negative	2977 (51.3)	457 (53.8)	365 (47.8)	1261 (53.9)	894 (48.4)	<0.001
Positive	2821 (48.7)	392 (46.2)	398 (52.2)	1077 (46.1)	954 (51.6)	

Abbreviations: BCS, breast‐conserving surgery; ER, estrogen receptor; IDC, infiltrating duct carcinoma; ILC, infiltrating lobular carcinoma; MAST, mastectomy; N, number; PR, progesterone receptor; RT, radiotherapy.

Patients with IDC (*p* < 0.001) and early T and N stage (*p* < 0.001) were more likely to receive BCS, while patients with poorly differentiated/undifferentiated (*p* = 0.001) and advanced T/N stage (*p* < 0.001) had more possibility to receive MAST. In addition, patients with younger age, advanced T/N stage, and ER/PR‐positive diseases were more likely to receive postoperative RT (Table 1).

### 3.2 Trends of local treatment receipt

Figure S1 shows the trends of different therapeutic modalities from 2004 to 2014. The proportions of receiving BCS decreased from 35.9% in 2004 to 26.2% in 2014 (*p* = 0.002), whereas RT receipt had no statistically significant tendency with 54.1% in 2004 and 53.8% in 2014 (*p* = 0.65) of the patients treated with BCS. In addition, the probability of patients receiving MAST increased from 64.1% in 2004 to 74.8% in 2014 (*p* = 0.002). However, there was no statistical significance in the probability of RT administration in patients treated with MAST, with 40.8% in 2004 and 46.6% in 2014 (*p* = 0.073), respectively.

### 3.3 Survival and prognostic analyses before PSM

**FIGURE 1:**
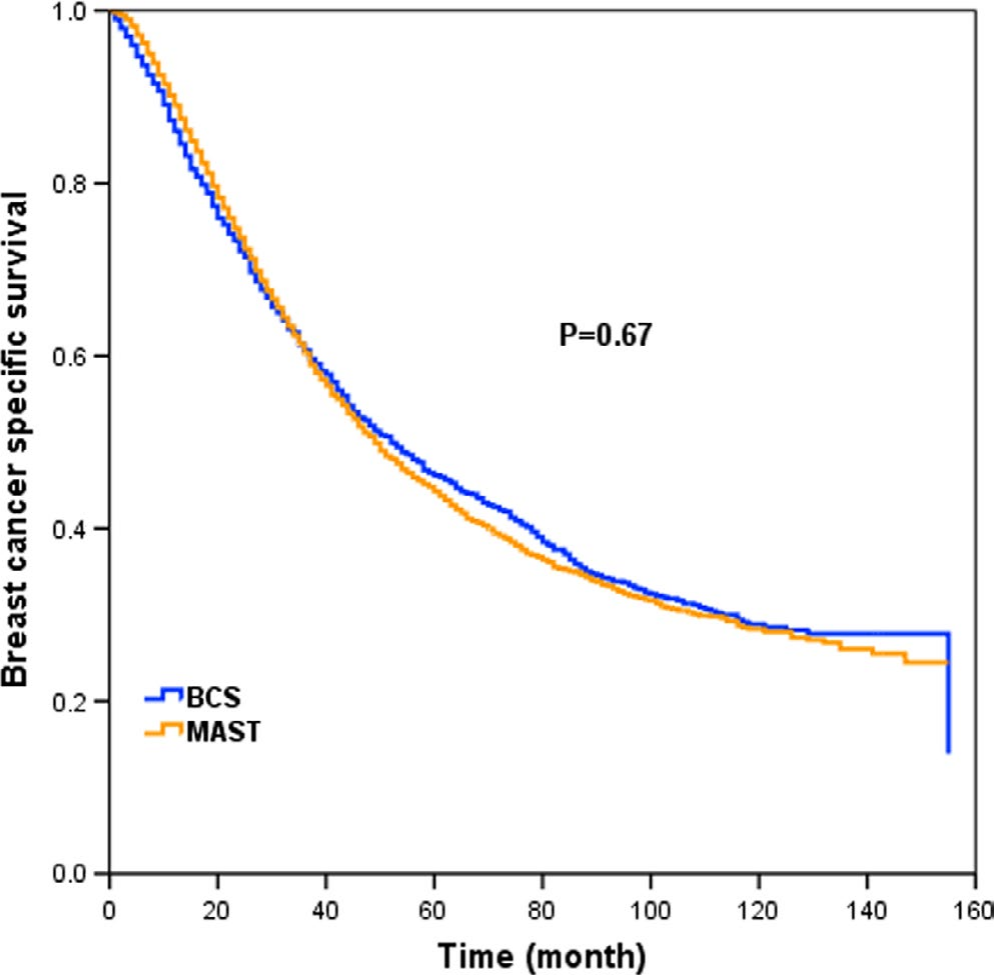
Breast cancer‐specific survival (BCSS) in patients treated with BCS and MAST for the whole cohort before propensity score matching (PSM)

**FIGURE 2:**
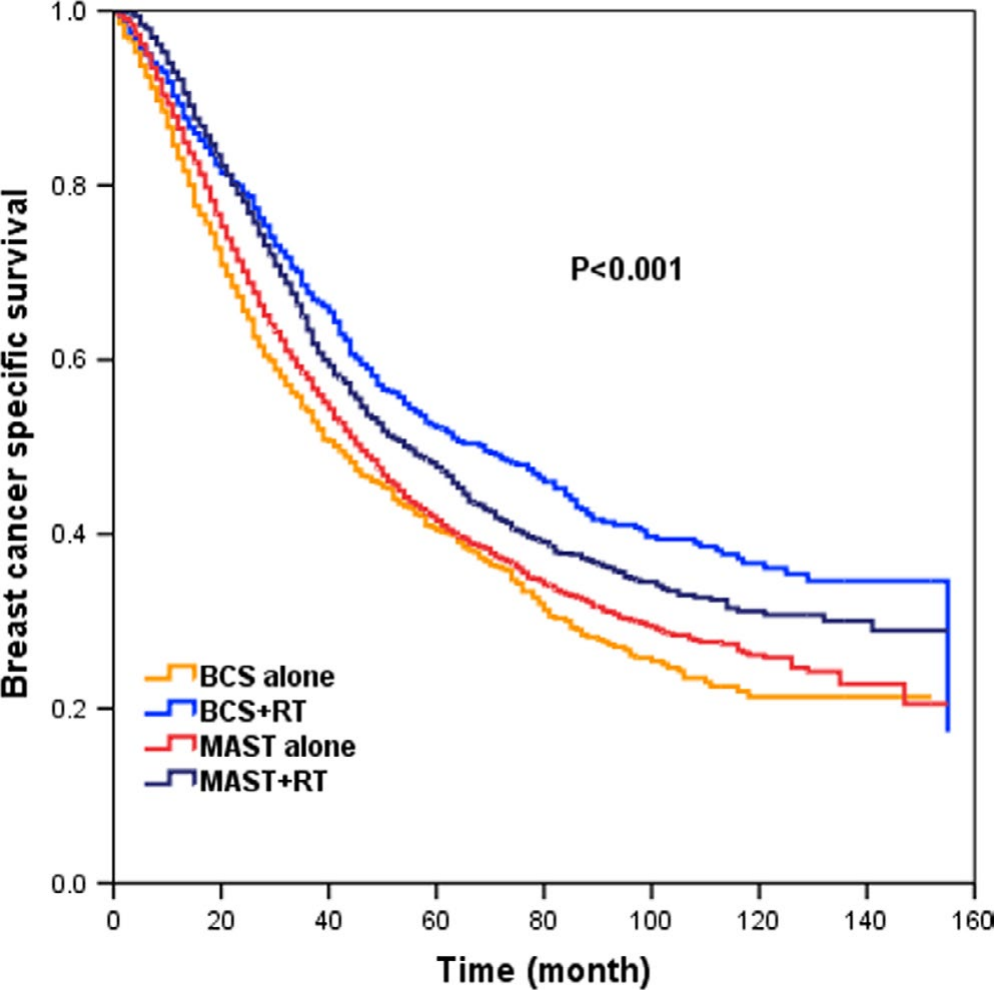
BCSS in patients receiving BCS alone, BCS+radiotherapy (RT), MAST alone, and MAST+RT for the whole cohort before PSM

With a median follow‐up time of 37 months (range, 0–155 months), a total of 3723 deaths occurred, including 3281 breast cancer‐specific deaths, and the 5‐year BCSS was 44.8%. The 5‐year BCSS was 46.2% and 44.3% (*p* = 0.67) between BCS and MAST groups, respectively (Figure 1). When further stratified by surgical approach and RT, the 5‐year BCSS was 40.5%, 52.3%, 41.5%, and 47.7% in patients treated with BCS alone, BCS+RT, MAST alone, and MAST+RT, respectively (*p* < 0.001) (Figure 2).

On multivariate Cox regression analysis for BCSS (Table 2), old age (≥65 years), non‐Hispanic black, ILC subtype, poorly differentiated/undifferentiated, and advanced T/N stage were independent adverse prognostic factors, while ER‐positive and PR‐positive were associated with favorable prognosis. In addition, patients receiving BCS+RT (hazard ratio [HR]: 0.701, 95% confidence interval [CI]: 0.614–0.8, *p* < 0.001), MAST alone (HR: 0.794, 95% CI: 0.716–0.88, *p* < 0.001), and MAST+RT (HR: 0.635, 95% CI: 0.569–0.71, *p* < 0.001) had better BCSS when using BCS as a reference.

**TABLE 2:** Multivariate analysis of cancer‐specific survival in patients with stage IV breast cancer before PSM

Variables	HR	95% CI	*p*
Age (years)			
<65	1		
≥65	1.14	1.052–1.236	0.001
Race/ethnicity			
Non‐Hispanic white	1		
Non‐Hispanic black	1.295	1.185–1.419	<0.001
Hispanic	0.988	0.887–1.100	0.825
Other	0.829	0.723–0.949	0.007
Pathological subtype			
IDC	1		
ILC	1.242	1.073–1.438	0.004
Other	1.023	0.932–1.124	0.632
Grade			
Well differentiated	1		
Moderately differentiated	1.147	0.943–1.394	0.169
Poorly differentiated/ undifferentiated	1.49	1.228–1.808	<0.001
Tumor stage			
T1	1		
T2	1.229	1.092–1.384	<0.001
T3	1.471	1.287–1.682	<0.001
T4	1.792	1.583–2.030	<0.001
Nodal stage			
N0	1		
N1	0.973	0.876–1.080	0.604
N2	1.058	0.941–1.190	0.347
N3	1.211	1.083–1.353	<0.001
ER status			
Negative	1		
Positive	0.774	0.703–0.852	<0.001
PR status			
Negative	1		
Positive	0.709	0.644–0.780	<0.001
Treatments			
BCS	1		
BCS+RT	0.701	0.614–0.800	<0.001
MAST	0.794	0.716–0.880	<0.001
MAST+RT	0.635	0.569–0.710	<0.001

Abbreviations: BCS, breast‐conserving surgery; CI, confidence interval; ER, estrogen receptor; HR, hazard ratio; IDC, infiltrating duct carcinoma; ILC, infiltrating lobular carcinoma; MAST, mastectomy; PR, progesterone receptor; RT, radiotherapy.

### 3.4 Survival and prognostic analyses after PSM

**FIGURE 3:**
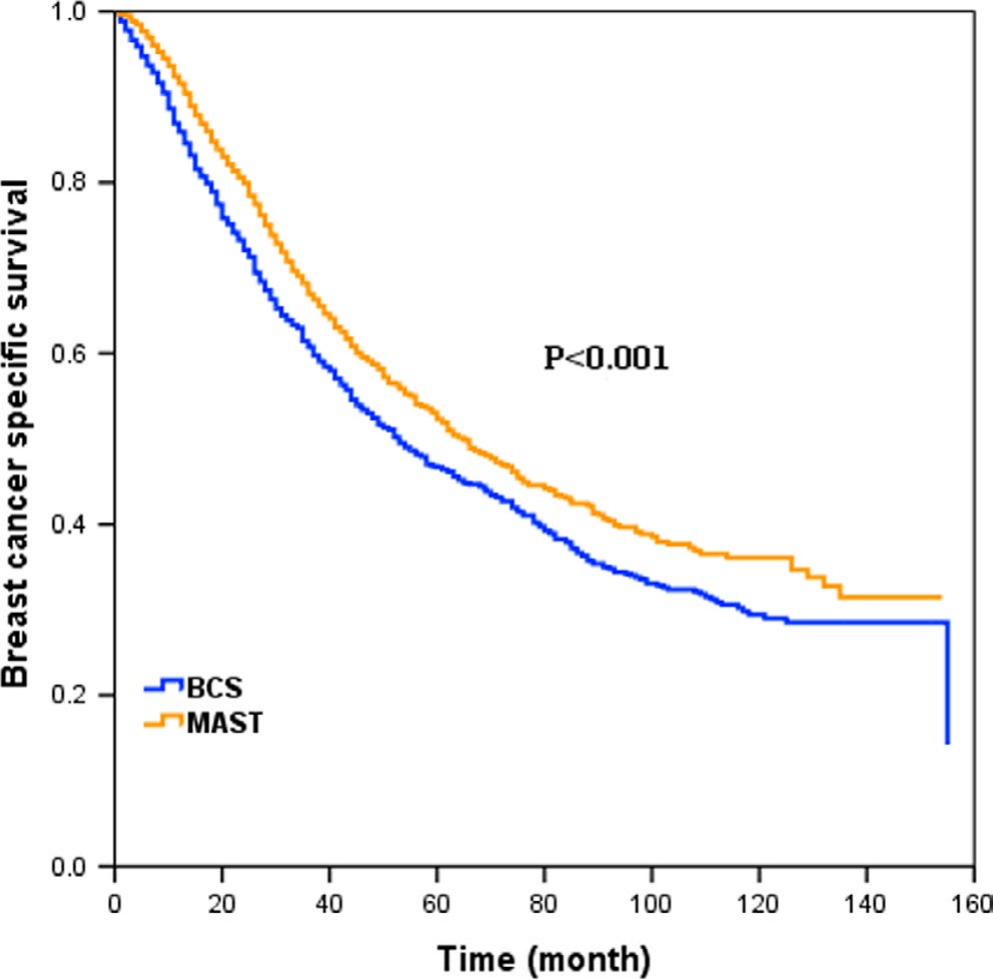
BCSS in patients treated with BCS and MAST for the whole patients after PSM

When performing PSM, a total of 1227 pairs were completely matched between BCS and MAST cohorts. The patient characteristics for the whole patients after PSM are shown in Table S1. After adjusting age, ethnicity, grade, histology, T/N stage, ER/PR status, patients who received BCS were associated with worse BCSS compared with those who received MAST in the whole cohort (HR: 0.82, 95% CI: 0.736–0.915, *p* < 0.001, Table S2). The survival curve is shown in Figure 3 (*p* < 0.001).

**FIGURE 4:**
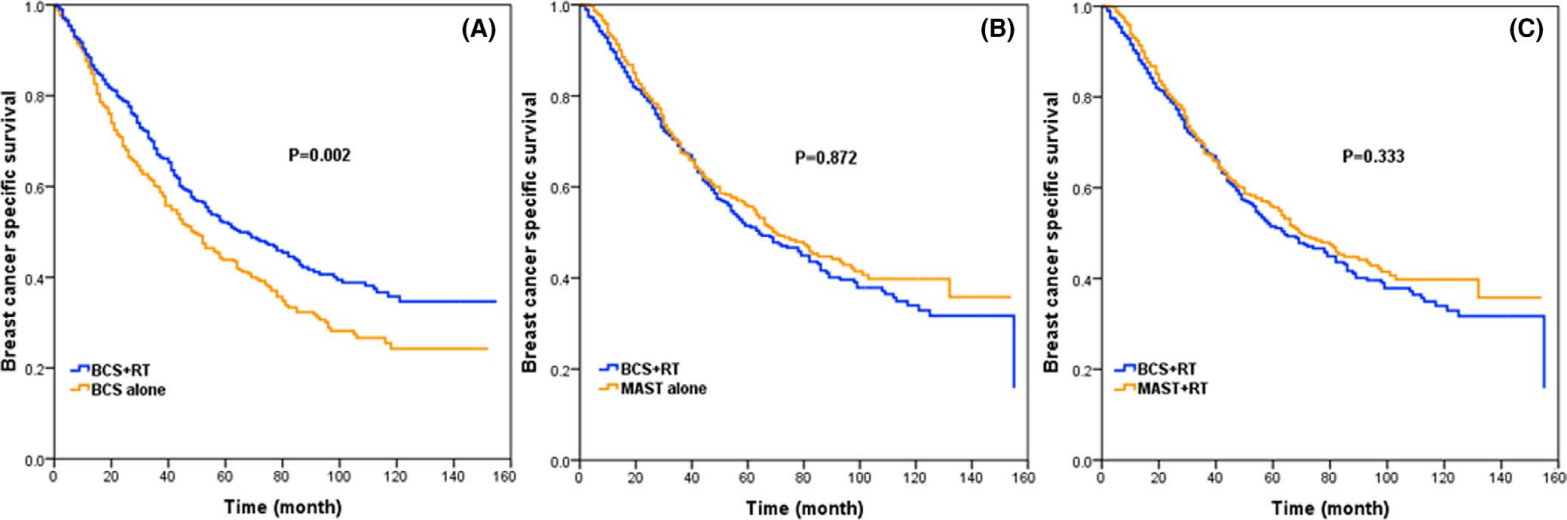
BCSS between BCS alone and BCS+RT groups (A), BCS+RT and MAST alone groups (B), and BCS+RT and MAST+RT groups (C) after PSM

When further stratifying by surgery and RT, 415, 578, and 464 pairs were completely matched between BCS alone and BCS+RT, BCS+RT and MAST alone, and BCS+RT and MAST+RT groups, respectively. The patient characteristics in the three groups are shown in Tables S3–S5, respectively. After adjusting age, ethnicity, grade, histology, T/N stage, ER/PR status, the 5‐year BCSS rates in patients receiving BCS alone and BCS+RT were 43.9% and 52.1%, respectively (*p* = 0.002, Figure 4A). Patients treated with BCS+RT had comparable survival compared with MAST alone group (51.3% and 50.1%, *p* = 0.872, Figure 4B), and MAST+RT group (51.5% and 55.7%, *p* = 0.333, Figure 4C). In multivariate analysis, patients treated with BCS alone had poorer BCSS than those treated with BCS+RT (HR: 0.732, 95% CI: 0.609–0.880, *p* < 0.001) (Table 3). However, there were comparable BCSS between BCS+RT and MAST alone groups (HR: 1.103, 95% CI: 0.862–1.191, *p* = 0.875, Table 4), and BCS+RT and MAST+RT groups (HR: 0.897, 95% CI: 0.747–1.078, *p* = 0.247) (Table 5).

**TABLE 3:** Multivariate analysis of BCSS in patients with stage IV breast cancer after PSM matched between BCS alone and BCS+RT groups

Variables	HR	95% CI	*p*
Age (years)			
<65	1		
≥65	0.798	0.619–1.029	0.082
Race/ethnicity			
Non‐Hispanic White	1		
Non‐Hispanic Black	1.171	0.893–1.536	0.253
Hispanic	0.809	0.596–1.098	0.174
Other	1.058	0.653–1.715	0.818
Pathological subtype			
IDC	1		
ILC	1.793	0.871–3.689	0.113
Other	0.99	0.691–1.420	0.958
Grade			
Well differentiated	1		
Moderately differentiated	1.58	0.798–3.127	0.19
Poorly differentiated/ undifferentiated	1.87	0.938–3.731	0.076
Tumor stage			
T1	1		
T2	1.267	0.996–1.611	0.054
T3	1.667	1.124–2.473	0.11
T4	1.809	1.285–2.545	<0.001
Nodal stage			
N0	1		
N1	1.016	0.809–1.275	0.891
N2	1.03	0.764–1.387	0.847
N3	0.997	0.734–1.354	0.984
ER status			
Negative	1		
Positive	0.82	0.608–1.106	0.194
PR status			
Negative	1		
Positive	0.659	0.492–0.883	0.005
Treatment			
BCS	1		
BCS+RT	0.732	0.609–0.88	<0.001

Abbreviations: BCS, breast‐conserving surgery; CI, confidence interval; ER, estrogen receptor; HR, hazard ratio; IDC, infiltrating duct carcinoma; ILC, infiltrating lobular carcinoma; PR, progesterone receptor; RT, radiotherapy.

**TABLE 4:** Multivariate analysis of BCSS in patients with de novo stage IV breast cancer after PSM matched between BCS+RT and MAST alone groups

Variables	HR	95% CI	*p*
Age (years)			
<65	1		
≥65	1.035	0.823–1.301	0.77
Race/ethnicity			
Non‐Hispanic White	1		
Non‐Hispanic Black	1.28	1.025–1.597	0.029
Hispanic	0.873	0.661–1.154	0.341
Other	0.837	0.590–1.189	0.321
Pathological subtype			
IDC	1		
ILC	1.505	0.984–2.304	0.059
Other	0.944	0.7–1.273	0.704
Grade			
Well differentiated	1		
Moderately differentiated	1.78	0.960–3.300	0.067
Poorly differentiated/ undifferentiated	2.242	1.208–4.163	0.011
Tumor stage			
T1	1		
T2	1.126	0.888–1.429	0.328
T3	1.619	1.178–2.225	0.003
T4	1.957	1.470–2.606	<0.001
Nodal stage			
N0	1		
N1	1.181	0.944–1.477	0.146
N2	1.296	0.979–1.715	0.07
N3	1.249	0.962–1.621	0.096
ER status			
Negative	1		
Positive	0.934	0.724–1.203	0.596
PR status			
Negative	1		
Positive	0.622	0.485–0.798	<0.001
Treatments			
BCS	1		
MAST+RT	1.013	0.862–1.191	0.875

Abbreviations: BCS, breast‐conserving surgery; CI, confidence interval; ER, estrogen receptor; HR, hazard ratio; IDC, infiltrating duct carcinoma; ILC, infiltrating lobular carcinoma; MAST, mastectomy; PR, progesterone receptor, RT, radiotherapy.

**TABLE 5:** Multivariate analysis of BCSS in patients with de novo stage IV breast cancer after PSM matched between BCS+RT and MAST+RT groups

Variables	HR	95% CI	*p*
Age			
<65	1		
≥65	1.06	0.801–1.402	0.683
Race/ethnicity			
Non‐Hispanic white	1		
Non‐Hispanic black	1.326	1.028–1.711	0.3
Hispanic	1.194	0.883–1.614	0.249
Other	0.842	0.580–1.221	0.364
Pathological subtype			
IDC	1		
ILC	1.471	0.859–2.520	0.16
Other	0.955	0.692–1.316	0.776
Grade			
Well differentiated	1		
Moderately differentiated	1.446	0.662–3.158	0.355
Poorly differentiated/ undifferentiated	2.016	0.920–4.415	0.08
Tumor stage			
T1	1		
T2	1.013	0.823–1.477	0.513
T3	1.211	0.833–1.76	0.316
T4	1.537	1.104–2.141	0.11
Nodal stage			
N0	1		
N1	1.033	0.766–1.393	0.831
N2	0.832	0.585–1.183	0.305
N3	1.137	0.824–1.569	0.434
ER status			
Negative	1		
Positive	0.858	0.648–1.137	0.286
PR status			
Negative	1		
Positive	0.651	0.49–0.865	0.003
Treatments			
BCS+RT	1		
MAST+RT	0.897	0.747–1.078	0.247

Abbreviations: BCS, breast‐conserving surgery; CI, confidence interval; ER, estrogen receptor; HR, hazard ratio; IDC, infiltrating duct carcinoma; ILC, infiltrating lobular carcinoma; MAST, mastectomy; PR, progesterone receptor; RT, radiotherapy.

## 4 DISCUSSION

In the present study, we aimed to assess the effect of surgical approaches and additional RT on patients with de novo stage IV breast cancer, and we found that BCS alone was significantly associated with poorer survival than BCS+RT, while additional RT to BCS achieved comparable BCSS compared with RT to MAST and MAST alone.

Traditionally, the primary therapeutic tactics in de novo stage IV breast cancer focus on systemic therapy; however, the application status of LRT including local surgery and RT remains unclear. In previous studies, 35–77.6% of the patients were treated with LRT, and of the patients receiving local surgery, approximately 12–51.4% received BCS, while 48.6–87% were treated with MAST.^7^, ^13^, ^14^, ^17^ However, the distribution of receiving RT had a significant difference in de novo stage IV breast cancer. In the study by Soran *et al*., postoperative RT was administrated to all BCS patients, while only 38% of the patients were treated with postoperative RT in the MAST cohort.^7^ In another two studies, 10.8% and 20.7% of the patients received post‐surgery RT, respectively. However, the details of the relationship between surgical procedures and postoperative RT were not recorded in these two studies.^14^, ^17^ Therefore, there was no consensus regarding the operation patterns and RT selection in de novo stage IV breast cancer, and decisions to administrate LRT were generally made by each institution according to their treatment protocols. In our study, the probability of receiving MAST (72.2%) was similar to previous studies.^7^, ^13^, ^14^, ^17^ However, significant differences were found in RT administration with 13.2% and 31.9% of patients treated with BCS+RT and MAST+RT, respectively. It is noteworthy that patients who received BCS decreased by 9.7%, while the patients treated with MAST increased by 10.7% over time. However, there was no significance in the probability of receiving post‐surgery RT. The reasons for the changing trends are unclear, and a possible explanation is that systemic therapy has achieved the best long‐term survival, resulting in decreased BCS administration and stable post‐surgery RT receipt. In addition, patients receiving MAST had more advanced T/N stage and poorer grade, leading to increased MAST selection for improving BCSS rate.^18^

The effect of surgical procedures on survival outcomes in de novo stage IV breast cancer remains unclear. Numerous previous studies with large cohorts have suggested that LRT could improve survival in this disease.^13^, ^14^, ^15^, ^16^ However, all of them were retrospective studies, and few regarded the survival difference between BCS and MAST. A retrospective study identified 566 patients with metastatic breast cancer who received surgery and no surgery therapy, and patients treated with MAST were associated with an improved OS compared with those who received BCS (37% vs. 20%, *p* = 0.04).^24^ However, 34% of the MAST group had preoperative chemotherapy, while only 15% of the BCS cohort received chemotherapy in their study. Another study from the National Cancer Database showed that patients receiving BCS were associated with poorer 3‐year overall survival (27.7% vs. 31.8%) than those receiving MAST.^2^ Interestingly, a similar result was found in the above studies that both BCS groups were more likely to have positive margins than MAST groups (55% vs. 27% by Khan *et al*.) (26% vs. 3% by McGuire *et al*.).^2^, ^24^ According to a previous study, patients with positive margins had significantly unfavorable survival than those with negative margins (*p* < 0.001),^2^ which might be a relatively reasonable explanation that BCS had poor survival than MAST. The same trend was found in our study. Despite this, the result of our study is more convincing for a large sample size of 5798 patients and chemotherapy receipt in the whole cohort.

For LRT in de novo stage IV breast cancer, most studies focused on surgical treatment, while rare studies regarded the value of RT in this patient subset. Two retrospective studies had drawn conflicting conclusions,^17^, ^25^ and there are currently no prospective studies assessing the effect of RT in de novo stage IV breast cancer. A study by Le Scodan *et al*. showed a 3‐year OS benefit in the LRT cohort compared with the non‐LRT cohort (43.4% vs. 26.7%, *p* < 0.001), and 91% of the patients were treated with RT (RT alone: 81%, surgery followed by RT: 13%) in LRT group.^25^ However, the patients receiving RT had smaller tumor size, lower nodal burden, more bone‐only metastases, less visceral and brain metastases, and more chemotherapy and endocrine therapy receipt.^25^ Another retrospective analysis have found that patients treated with surgery alone had comparable local recurrence‐free survival or OS compared with surgery+RT.^26^ However, the details of lymph node (LN) status were not recorded in the surgery alone and surgery+RT cohort in their study, which could affect RT administration. In our population‐based study with a large sample size, additional RT to BCS or MAST both achieved better survival than surgery alone in the premise of receiving chemotherapy, which was similar to previous results.^18^, ^27^ The most important is that our data had detailed information of LN status with 80.5% and 86.3% had positive LNs in the surgery alone and surgery+RT cohort, respectively. In non‐metastatic breast cancer with positive LNs, post‐surgery RT could reduce the locoregional recurrence rate and improve survival.^28^, ^29^ Therefore, RT may also play an important role in LRT of metastatic breast cancer with positive LNs.

Advances in systemic management including taxane‐based chemotherapy, targeted therapy, and endocrine therapy allow patients to live longer with their disease.^5^, ^6^, ^7^ Quality of life in cancer patients is increasingly regarded as a clinically relevant goal that warrant consideration, and the advantage of smaller surgery and longer survival is gradually highlighted. As our study showed, BCS+RT had non‐ inferior survival than MAST+RT or surgery alone; therefore, additional RT to BCS could be the optimal choice in de novo stage IV breast cancer. There are many ascendancies regarding BCS+RT as the LRT patterns in stage IV breast cancer. Firstly, patients with BCS suffer less treatment‐related side effects such as inflammatory response and tissue damage than those with MAST.^30^ Secondly, patients treated with BCS have a better self‐image and sexual well‐being, leading to better psychological health and life satisfaction than those treated with MAST.^31^, ^32^ Thirdly, BCS procedures are more likely to be carried out by experienced surgeons in teaching hospitals with detailed discussion, which is associated with a better outcome. ^33^ Fourthly, patients receiving BCS have better treatment compliance and tolerance, and they may have more possibilities of receiving post‐surgery medical surveillance. Therefore, cooperative multidisciplinary care is highly significant in the management of de novo stage IV breast carcinoma, and the addition of RT to BCS might be the optimal treatment mode.

There are several limitations to acknowledge in our study. Firstly, the data were obtained from the SEER database, and selection biases that retrospective study inherently exists could not be ruled out, even though PSM analysis was used. Secondly, detailed treatment information including chemotherapy regimen, endocrine therapy, anti‐HER2 targeted therapies, RT technique, RT dose, target volume, and sequential surgery, chemotherapy, and RT data was unavailable in the SEER database. Fourthly, metastasis sites and treatment patterns after relapse were not recorded, which could also interfere with survival outcomes. The strong point of our study is that our study detailedly assessed the effect of BCS, MAST, and additional RT in de novo stage IV breast cancer based on a large population in the premise of chemotherapy, especially few scholars to explore the effect of surgical approach in this patient subset.

In conclusion, our study suggests that postoperative RT improves BCSS in patients with de novo stage IV breast cancer, and BCS+RT shows a non‐inferior outcome compared to MAST+RT. BCS+RT may be the optimal local management of de novo stage IV breast cancer. More studies are needed to confirm our results.

## CONFLICT OF INTERESTS

The authors declare that there is no conflict of interest.

## AUTHOR CONTRIBUTIONS

Jun Wang, Shi‐Ping Yang, Ping Zhou, San‐Gang Wu, and Zhen‐Yu He: Conceptualization, data curation, formal analysis, investigation, methodology, project administration, supervision, validation, visualization, writing–original draft, and writing–review and editing. Chen‐Lu Lian, Jian Lei, and Li Hua: Investigation, and writing–review and editing.

## Supporting information

Fig S1

Table S1‐S5

## Data Availability

The data sets generated for this study are available in the SEER database (https://seer.cancer.gov/about/overview.html).
