# Supplementary figures and images for: Additional radiotherapy to breast‐conserving surgery is an optional treatment for de novo stage IV breast cancer: A population‐based analysis
Source: Cancer Med. 2021 Feb 14;10(5):1634–43. doi: 10.1002/cam4.3751 (PMC7940213; doi:10.1002/cam4.3751)

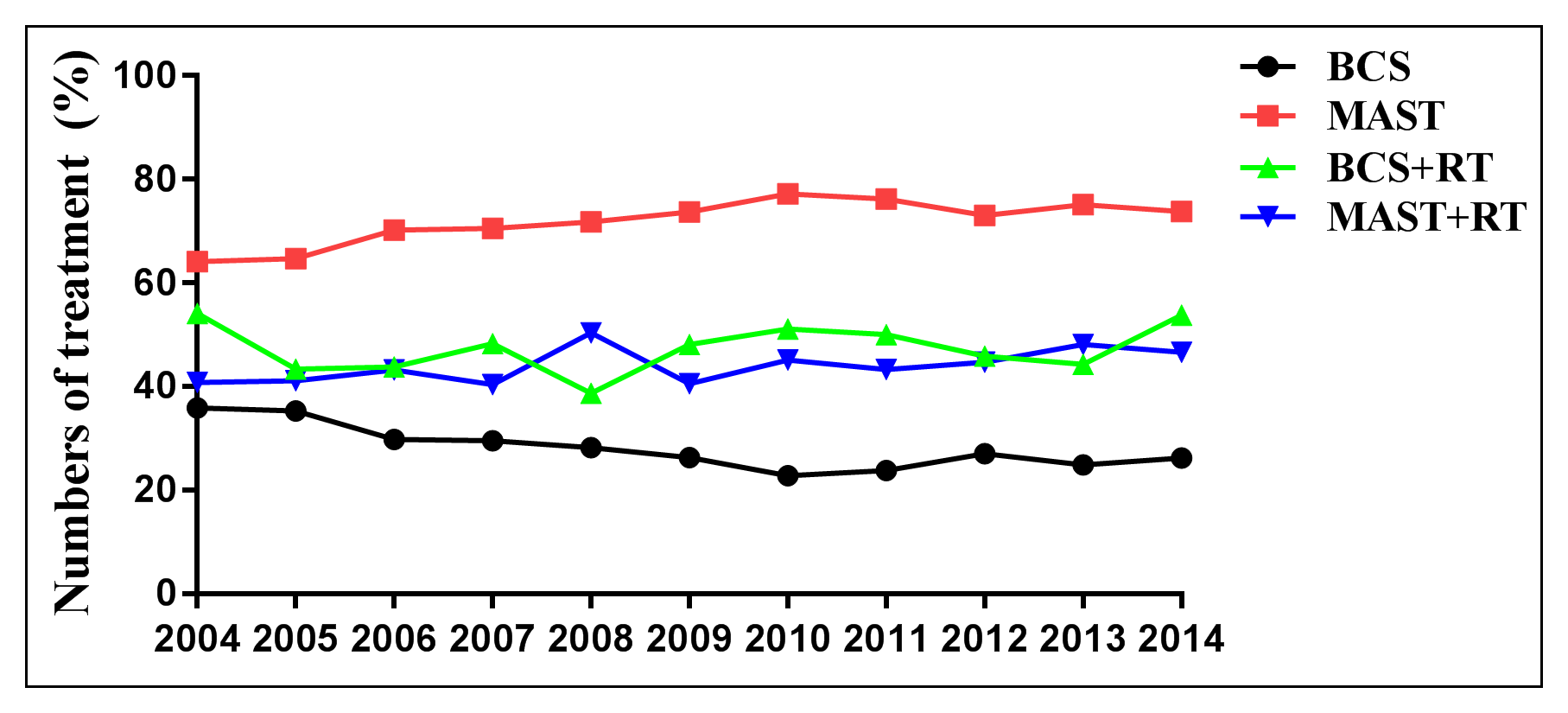

Supplement: Supplementary file 1 — Fig S1 [file CAM4-10-1634-s001.tif]
