# Supplementary material for: Additional radiotherapy to breast‐conserving surgery is an optional treatment for de novo stage IV breast cancer: A population‐based analysis
Source: Cancer Med. 2021 Feb 14;10(5):1634–43. doi: 10.1002/cam4.3751 (PMC7940213; doi:10.1002/cam4.3751)
Supplement: Supplementary file 2 — Table S1‐S5 [file CAM4-10-1634-s002.doc]

| **Table S1.** Patient baseline characteristics in *de novo* stage IV breast cancer after PSM matched between BCS and MAST groups | | | |
| --- | --- | --- | --- |
| **Variables** | **N(%)** | **BCS** | **MAST** |
| **Age (years)** |  |  |  |
| <65 | 1966(80.1) | 983(80.1) | 983(80.1) |
| ≥65 | 488(19.9) | 244(19.9) | 244(19.9) |
| **Race/ethnicity** |  |  |  |
| Non-Hispanic White | 1642(66.9) | 821(66.9) | 821(66.9) |
| Non-Hispanic Black | 384(15.6) | 192(15.6) | 192(15.6) |
| Hispanic | 264(10.8) | 132(10.8) | 132(10.8) |
| Other | 164(6.7) | 82(6.7) | 82(6.7) |
| **Pathological subtype** |  |  |  |
| IDC | 2106(85.8) | 1053(85.8) | 1053(85.8) |
| ILC | 88(3.6) | 44(3.6) | 44(3.6) |
| Other | 260(10.6) | 130(10.6) | 130(10.6) |
| **Grade** |  |  |  |
| Well differentiated | 82(3.3) | 41(3.3) | 41(3.3) |
| Moderately differentiated | 758(30.9) | 379(30.9) | 379(30.9) |
| Poorly differentiated/ undifferentiated | 1614(65.8) | 807(65.8) | 807(65.8) |
| **Tumor stage** |  |  |  |
| T1 | 436(17.8) | 218(17.8) | 218(17.8) |
| T2 | 1334(54.4) | 667(54.4) | 667(54.4) |
| T3 | 266(10.8) | 133(10.8) | 133(10.8) |
| T4 | 418(17) | 209(17) | 209(17) |
| **Nodal stage** |  |  |  |
| N0 | 500(20.4) | 250(20.4) | 250(20.4) |
| N1 | 1044(42.5) | 522(42.5) | 522(42.5) |
| N2 | 440(17.9) | 220(17.9) | 220(17.9) |
| N3 | 470(19.2) | 235(19.2) | 235(19.2) |
| **ER status** |  |  |  |
| Negative | 942(38.4) | 471(38.4) | 471(38.4) |
| Positive | 1512(61.6) | 756(61.6) | 756(61.6) |
| **PR status** |  |  |  |
| Negative | 1228(50) | 614(50) | 614(50) |
| Positive | 1226(50) | 613(50) | 613(50) |
| N, number; BCS, breast conserving surgery; MAST, mastectomy; IDC, infiltrating duct carcinoma; ILC, infiltrating lobular carcinoma; ER, estrogen receptor; PR, progesterone receptor. | | | |

| **Table S2.** Multivariate analysis of BCSS in the whole patients after PSM matched between BCS and MAST groups | | | |
| --- | --- | --- | --- |
| **Variables** | **HR** | **95%CI** | **P** |
| **Age** |  |  |  |
| <65 | 1 |  |  |
| ≥65 | 1.19 | 1.042-1.367 | 0.011 |
| **Race/ethnicity** |  |  |  |
| Non-Hispanic white | 1 |  |  |
| Non-Hispanic black | 1.278 | 1.1-1.484 | 0.001 |
| Hispanic | 1.032 | 0.863-1.235 | 0.728 |
| Other | 0.856 | 0.676-1.083 | 0.195 |
| **Pathological subtype** |  |  |  |
| IDC | 1 |  |  |
| ILC | 1.436 | 1.062-1.94 | 0.019 |
| Other | 1.111 | 0.934-1.322 | 0.234 |
| **Grade** |  |  |  |
| Well differentiated | 1 |  |  |
| Moderately differentiated | 1.291 | 0.873-1.907 | 0.2 |
| Poorly differentiated/ undifferentiated | 1.686 | 1.141-2.492 | 0.009 |
| **Tumor stage** |  |  |  |
| T1 | 1 |  |  |
| T2 | 1.193 | 1.011-1.406 | 0.036 |
| T3 | 1.511 | 1.22-1.871 | <0.001 |
| T4 | 1.934 | 1.597-2.343 | <0.001 |
| **Nodal stage** |  |  |  |
| N0 | 1 |  |  |
| N1 | 1.018 | 0.875-1.185 | 0.815 |
| N2 | 1.071 | 0.892-1.285 | 0.462 |
| N3 | 1.208 | 1.015-1.438 | 0.033 |
| **ER status** |  |  |  |
| Negative | 1 |  |  |
| Positive | 0.811 | 0.683-0.963 | 0.017 |
| **PR status** |  |  |  |
| Negative | 1 |  |  |
| Positive | 0.666 | 0.561-0.792 | <0.001 |
| **Treatments** |  |  |  |
| BCS | 1 |  |  |
| MAST | 0.82 | 0.736-0.915 | <0.001 |
| HR, hazard ratio; CI, confidence interval; IDC, infiltrating duct carcinoma; ILC, infiltrating lobular carcinoma; ER, estrogen receptor; PR, progesterone receptor; BCS, breast conserving surgery; MAST, mastectomy. | | | |

| **Table S3.** Patient baseline characteristics in *de novo* stage IV breast cancer after PSM matched between BCS alone and BCS+RT groups | | | |
| --- | --- | --- | --- |
| **Variables** | **N(%)** | **BCS** | **BCS+RT** |
| **Age (years)** |  |  |  |
| <65 | 698(84.1) | 349(84.1) | 349(84.1) |
| ≥65 | 132(15.9) | 66(15.9) | 66(15.9) |
| **Race/ethnicity** |  |  |  |
| Non-Hispanic White | 586(70.6) | 293(70.6) | 293(70.6) |
| Non-Hispanic Black | 118(14.2) | 59(14.2) | 59(14.2) |
| Hispanic | 92(11.1) | 46(11.1) | 46(11.1) |
| Other | 34(4.1) | 17(4.1) | 17(4.1) |
| **Pathological subtype** |  |  |  |
| IDC | 758(91.3) | 379(91.3) | 379(91.3) |
| ILC | 14(1.7) | 7(1.7) | 7(1.7) |
| Other | 58(7) | 29(7) | 29(7) |
| **Grade** |  |  |  |
| Well differentiated | 32(3.9) | 16(3.9) | 16(3.9) |
| Moderately differentiated | 240(28.9) | 120(28.9) | 120(28.9) |
| Poorly differentiated/ undifferentiated | 558(67.2) | 279(67.2) | 279(67.2) |
| **Tumor stage** |  |  |  |
| T1 | 208(25.1) | 104(25.1) | 104(25.1) |
| T2 | 482(58.1) | 241(58.1) | 241(58.1) |
| T3 | 54(6.5) | 27(6.5) | 27(6.5) |
| T4 | 86(10.4) | 43(10.4) | 43(10.4) |
| **Nodal stage** |  |  |  |
| N0 | 252(30.4) | 126(30.4) | 126(30.4) |
| N1 | 336(40.5) | 168(40.5) | 168(40.5) |
| N2 | 130(15.7) | 65(15.7) | 65(15.7) |
| N3 | 112(13.5) | 56(13.5) | 56(13.5) |
| **ER status** |  |  |  |
| Negative | 318(38.3) | 159(38.3) | 159(38.3) |
| Positive | 512(61.7) | 256(61.7) | 256(61.7) |
| **PR status** |  |  |  |
| Negative | 412(49.6) | 206(49.6) | 206(49.6) |
| Positive | 418(50.4) | 209(50.4) | 209(50.4) |
| N, number; BCS, breast conserving surgery; RT, radiotherapy; IDC, infiltrating duct carcinoma; ILC, infiltrating lobular carcinoma; ER, estrogen receptor; PR, progesterone receptor, | | | |

| **Table S4.** Patient baseline characteristics in *de novo* stage IV breast cancer after PSM matched between BCS+RT and MAST groups | | | |
| --- | --- | --- | --- |
| **Variables** | **N(%)** | **BCS+RT** | **MAST** |
| **Age (years)** |  |  |  |
| <65 | 980(84.8) | 490(84.8) | 490(84.8) |
| ≥65 | 176(15.2) | 88(15.2) | 88(15.2) |
| **Race/ethnicity** |  |  |  |
| Non-Hispanic White | 782(67.6) | 391(67.6) | 391(67.6) |
| Non-Hispanic Black | 176(15.2) | 88(15.2) | 88(15.2) |
| Hispanic | 116(10) | 58(10) | 58(10) |
| Other | 82(7.1) | 41(7.1) | 41(7.1) |
| **Pathological subtype** |  |  |  |
| IDC | 1020(88.2) | 510(88.2) | 510(88.2) |
| ILC | 42(3.6) | 21(3.6) | 21(3.6) |
| Other | 94(8.1) | 47(8.1) | 47(8.1) |
| **Grade** |  |  |  |
| Well differentiated | 38(3.3) | 19(3.3) | 19(3.3) |
| Moderately differentiated | 374(32.4) | 187(32.4) | 187(32.4) |
| Poorly differentiated/ undifferentiated | 744(64.4) | 372(64.4) | 372(64.4) |
| **Tumor stage** |  |  |  |
| T1 | 220(19) | 110(19) | 110(19) |
| T2 | 650(56.2) | 325(56.2) | 325(56.2) |
| T3 | 112(9.7) | 56(9.7) | 56(9.7) |
| T4 | 174(15.1) | 87(15.1) | 87(15.1) |
| **Nodal stage** |  |  |  |
| N0 | 260(22.5) | 130(22.5) | 130(22.5) |
| N1 | 488(42.2) | 244(42.2) | 244(42.2) |
| N2 | 184(15.9) | 92(15.9) | 92(15.9) |
| N3 | 224(19.4) | 112(19.4) | 112(19.4) |
| **ER status** |  |  |  |
| Negative | 420(36.3) | 210(36.3) | 210(36.3) |
| Positive | 736(63.7) | 368(63.7) | 368(63.7) |
| **PR status** |  |  |  |
| Negative | 564(48.8) | 282(48.8) | 282(48.8) |
| Positive | 592(51.2) | 296(51.2) | 296(51.2) |
| N, number; BCS, breast conserving surgery; RT, radiotherapy; MAST, mastectomy; IDC, infiltrating duct carcinoma; ILC, infiltrating lobular carcinoma; ER, estrogen receptor;; PR, progesterone receptor. | | | |

| **Table S5.** Patient baseline characteristics in *de novo* stage IV breast cancer after PSM matched between BCS+RT and BCS+RT groups | | | |
| --- | --- | --- | --- |
| **Variables** | **N(%)** | **BCS+RT** | **MAST+RT** |
| **Age (years)** |  |  |  |
| <65 | 810(87.3) | 405(87.3) | 405(87.3) |
| ≥65 | 118(12.7) | 59(12.7) | 59(12.7) |
| **Race/ethnicity** |  |  |  |
| Non-Hispanic White | 608(65.5) | 304(65.5) | 304(65.5) |
| Non-Hispanic Black | 144(15.5) | 72(15.5) | 72(15.5) |
| Hispanic | 100(10.8) | 50(10.8) | 50(10.8) |
| Other | 76(8.2) | 38(8.2) | 38(8.2) |
| **Pathological subtype** |  |  |  |
| IDC | 806(86.9) | 403(86.9) | 403(86.9) |
| ILC | 30(3.2) | 15(3.2) | 15(3.2) |
| Other | 92(9.9) | 46(9.9) | 46(9.9) |
| **Grade** |  |  |  |
| Well differentiated | 22(2.4) | 11(2.4) | 11(2.4) |
| Moderately differentiated | 294(31.7) | 147(31.7) | 147(31.7) |
| Poorly differentiated/ undifferentiated | 612(65.9) | 306(65.9) | 306(65.9) |
| **Tumor stage** |  |  |  |
| T1 | 140(15.1) | 70(15.1) | 70(15.1) |
| T2 | 514(55.4) | 257(55.4) | 257(55.4) |
| T3 | 108(11.6) | 54(11.6) | 54(11.6) |
| T4 | 166(17.9) | 83(17.9) | 83(17.9) |
| **Nodal stage** |  |  |  |
| N0 | 166(12.5) | 58(12.5) | 58(12.5) |
| N1 | 434(46.8) | 217(46.8) | 217(46.8) |
| N2 | 168(18.1) | 84(18.1) | 84(18.1) |
| N3 | 210(22.6) | 105(22.6) | 105(22.6) |
| **ER status** |  |  |  |
| Negative | 324(34.9) | 162(34.9) | 162(34.9) |
| Positive | 604(65.1) | 302(65.1) | 302(65.1) |
| **PR status** |  |  |  |
| Negative | 434(46.8) | 217(46.8) | 217(46.8) |
| Positive | 494(53.2) | 247(53.2) | 247(53.2) |
| N, number; BCS, breast conserving surgery; RT, radiotherapy; IDC, infiltrating duct carcinoma; ILC, infiltrating lobular carcinoma; ER, estrogen receptor; PR, progesterone receptor. | | | |
